# Adhesive Joint Degradation Due to Hardener-to-Epoxy Ratio Inaccuracy under Varying Curing and Thermal Operating Conditions

**DOI:** 10.3390/ma15217765

**Published:** 2022-11-03

**Authors:** Jakub Szabelski, Robert Karpiński, Józef Jonak, Mariaenrica Frigione

**Affiliations:** 1 Department of Computerisation and Production Robotisation, Faculty of Mechanical Engineering, Lubli University of Technology, Nadbystrzycka 36, 20-618 Lublin, Poland; 2 Department of Innovation Engineering, University of Salento, Provinciale Lecce-Monteroni, 73100 Lecce, Italy; 3 Department of Machine Design and Mechatronics, Faculty of Mechanical Engineering, Lublin University of Technology, Nadbystrzycka 36, 20-618 Lublin, Poland

**Keywords:** epoxy adhesives, adhesive joint, degradation, heat curing, thermal stability, resin hardener mix ratio, mathematical modelling

## Abstract

This paper presents the results of an experimental study of adhesive joint strength with consideration of the inaccuracy of the hardener dosage, in the context of evaluating the degradation of joints when used either at ambient or elevated temperatures. The butt joint strength characteristics were assessed for two types of adhesives—rigid and flexible—and two curing scenarios—with and without heat curing. An excess hardener was shown to be significantly more unfavourable than its deficiency, which can ultimately be considered as a recommendation for forming epoxy adhesive joint assemblies. In order to fully understand the relationship between the analysed mechanical properties of the material and the influence of component ratio excesses and heating, a process of fitting basic mathematical models to the obtained experimental data was also performed.

## 1. Introduction

**Figure 1:**
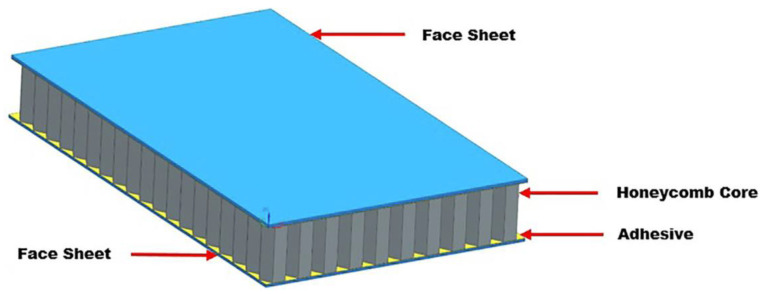
3D model of honeycomb sandwich structure.

Adhesives have been known to man for thousands of years. Initially, natural materials based, for example, on birch tar, bituminous substances (asphalts) or animal collagen derivatives were used, but the constant advance of knowledge in the fields of chemistry, physics, materials engineering and mechanics itself has led to the development of this technique as well [1]. Today, thousands of adhesive compositions are commercially available. There are adhesives dedicated to specific substrates, specific operating conditions and joint loading conditions, as well as universal adhesives. We can find adhesives for high-strength joints, requiring precise steps of the bonding operation (preparation of the surface, the adhesive, the joint curing method), and adhesives for detachable joints, e.g., light paper adhesives, adhesives neutralised by solvents or heat. Today, a wide variety of materials are bonded using this technique, from tissues (adhesives used in biomedical engineering) and simple construction materials to even the most complex manufacture of honeycomb composites used in the aerospace industry (Figure 1 [2]). This shows how important the adhesive bonding operation is today. Technologists are using it more frequently today not only as a complementary solution for joining materials, as in certain cases, adhesive bonding may be the only possible joint forming method [3]. These joint types are successively substituting traditional joining methods such as welding, soldering and bolted joints. The reason for this is that adhesive joints have several significant advantages over classic material joining techniques. These include the simplicity of manufacture, no increase in the weight of the joined elements, universality (many materials that are difficult to join using other methods can be joined) and cost (it is usually the cheapest method of making connections. Wherever the typical disadvantages of joining are not disqualifying for the use of this method, it can be a serious competitor to existing techniques. Among the biggest disadvantages are: the joint strength, which is difficult to calculate and often requires destructive experimental trials, the bonding time, the thermal resistance and the need for a proper and precise execution of the entire bonding process. Therefore, much attention is paid to the analysis of the adhesive joint strength and the testing of factors that can ensure an increase in the quality and functional properties of the joint.

The final strength of structural adhesive joints depends on many factors, both technological and structural [4,5]. The most important ones, the optimisation of which can significantly increase the strength of structural adhesive joints, include the method of preparing the surfaces of the elements to be joined (achieving an optimal surface condition through mechanical, electrochemical or chemical treatment operations) [6,7]. Another important issue is the selection of a proper adhesive for joining specific surfaces and the modification of the adhesive composition (introducing fillers, plasticisers, etc.) [8,9,10,11,12]. The conditions of preparing the joints also play a significant role in obtaining strong and reliable joints [13,14], as well as the manufacturing technology, taking into account the geometry of the joint, the method of loading the joint and the conditions of curing the adhesive joint [15,16,17]. The strength of the adhesive bond is also affected by various types of defects in the form of non-joined areas. They can be caused, for example, by air bubbles in the adhesive, which are the result of mixing too much of the two-component adhesive too quickly, or by physical impurities in the adhesive. Such an adhesive will have a porous structure after cross-linking, with the risk of local voids at the interface between the bonding material and the adhesive, which will impair adhesion, and voids in the internal structure of the adhesive, which will weaken the cohesion of the adhesive material [18,19].

Typical defects in adhesive bonds include damage [20]:

(1) adherend
  - failure of one or both adherends—substrate failure,
  - failure of an adherend—cohesive substrate failure,
  - failure through lamination—delamination failure,
(2) adhesive
  - Cohesion failure
    - Cohesion failure
    - Special cohesion failure
    - Failure with stress whitening of adhesive
  - Adhesion failure
  - Adhesion and cohesion failure with peel
(3) Corrosion at the interface—debonding due to bondline corrosion.

Taking into consideration the wide variety of adhesive compositions, the large quantitative parameters and the vast range of parameters that need to be taken into account when producing the strongest possible joints, any tests carried out on this subject are complex and require considerable time and financial expenditure, as, very often, the final strength of the finished joint is verified by destructive testing. The analysis of changes in mechanical parameters is extremely important for the estimation of the service life of adhesive bonds and in the design of modern materials. Conducting a large number of destructive experimental tests is often not possible due to time and economic constraints. The methods for solving such a problem can include the implementation of mathematical modelling [21,22], computer methods such as the finite element method (FEM) [23,24,25,26,27,28], the boundary element method (BEM) [29,30], the application of predictive models [31,32,33], machine learning methods [33,34,35,36] and analytical data analysis [37,38]. The models developed by this approach allow the most important relationships between individual parameters and mechanical properties to be determined. Using previously acquired experimental data, they can also indicate the most promising research direction and help to minimise the number of physical tests performed, thereby significantly reducing the cost and time of research.

The research analysed the problem of joints made with adhesives of a very popular type—epoxies, produced by the chemical reaction of the polymerisation of an epoxy resin combined with a hardener. Adhesives of this type, as they require two different components, are usually supplied as commercial compositions in a separated form, although there also exist single-component solutions, where the components are already mixed but require activation, e.g., by temperature or UV light. It is also possible to develop custom compositions by reacting one of the many available epoxy resins with selected hardeners (e.g., amine). When preparing adhesives (as well as other, two-component polymeric materials [39,40,41,42]), the correct stoichiometric ratio of the curing resin and hardener is crucial. This can determine not only the strength of the adhesive bond but many other performance parameters such as the resistance to heat, harmful environmental influences, oils, etc. The qualitative impact of the inaccuracy of the ratio of the components of an epoxy adhesive on these characteristics does not seem to have been fully analysed, especially when combined with consideration of the other two factors [43], and additional research expanding the range of tested adhesives by adhesives of different properties seems to be justified. The heat role mentioned earlier, as an initiator of the polymerisation reaction of single-component epoxy adhesives, can also be used in the reaction of typical two-component adhesives not only as a reaction accelerator but also as something that can allow a higher degree of polymerisation to be achieved and thus create stronger bonds.

In view of the above-described problems of bonding defects related to the methods of their prevention, attention was paid to the aspect of the potential degradation of adhesive bonds as a result of the combination of harmful effects of temperature during bond operation and, at the same time, inaccuracy in the technological production of the adhesive (epoxy–resin–amine–hardener ratio), taking into account the technology of bond curing (cold/heat curing). Although some degradation of bond strength with increasing temperatures and some degradation related to the inaccuracy of the above-mentioned ratio can be assumed, it is not entirely certain what effect the curing conditions will have on the degree of degradation by the above-mentioned factors. Therefore, this study set out to determine the degree of adhesive joint strength degradation with an inaccurate component ratio of the hardener in the epoxy adhesive and to evaluate this degradation when heat-cured joints are operated at ambient and elevated temperatures for rigid and elastic adhesives. This will allow general recommendations to be formulated for the use of heat curing operations when there is uncertainty in the adhesive composition and may help to ensure greater adhesive joint assembly durability at elevated temperatures.

## 2. Materials and Methods

### 2.1. Materials and Sample Preparation

The research involved testing adhesive butt joints prepared using two adhesives that differed in their parameters: a commercial Loctite Hysol 9492 composition and an adhesive based on Epidian 57 epoxy resin and a PAC hardener. Both are two-component, chemically curing epoxy formulations with similar curing times and strengths of the joints formed. The most important difference between joints made with the two above-mentioned adhesives is that they will differ significantly in stiffness after curing. Hysol 9492 is more rigid, with the Young’s modulus being approximately 5× that of the PAC-crosslinked Epidian 57.

Hysol 9492 is an epoxy adhesive belonging to the wide range of Loctite-brand adhesives. It is supplied in double cartridges with an applicator (mixer) at the tip and is designed to mix the components, in the recommended volumetric proportion, as soon as it leaves the applicator. The volumetric ratio of parts A to B (resin to hardener) is 2:1. It is a multi-purpose adhesive and can be used in many different applications in addition to its function as a traditional adhesive—e.g., as a sealant or for repairing materials (e.g., removing pores and irregularities from the surface of castings or forgings).

Epidian 57 epoxy resin is a clear, yellow, viscous liquid that is produced by the reaction of bisphenol A and epichlorohydrin, modified with unsaturated polyester resin. In addition to its traditional application, i.e., making adhesive bonds, the resin is also used to produce, in combination with glass fibre, high-strength glass-epoxy laminates. For the curing of Epidian 57, the hardener used in the study was PAC, an amber-coloured viscous liquid consisting of approx. 8–12% triethylenetetramine and enriched with unsaturated fatty acids. The ratio range recommended by the manufacturer for the curing of Epidian 57 is 100:50–80.

The choice of adhesive materials was driven by an attempt to test materials with differing properties. Typically, flexible adhesives are more resistant to elongation but can withstand less stress. The effect of the other parameters examined in the paper on the final strength of the joint may be also different for rigid adhesives and different for flexible ones. Modifying the amount of the hardener, the research analysed joints produced by adhesives made with a deficiency and with an excess of the hardener, in the combinations of −50%, −30%, −10%, +10%, +30% and in the ratio that is considered appropriate (2:1 for both adhesives, i.e., 0% inaccuracy). Also included in the test plan were the curing conditions of the bond, the effect of which was also studied in this work, i.e., according to the manufacturers’ recommendations: long-term curing at ambient temperature (25 °C: 7 days for E57/PAC and 3 days for Hysol 9492) and heat curing (100 °C: 2 h for E57/PAC and 1 h for Hysol 9492).

**Figure 2:**
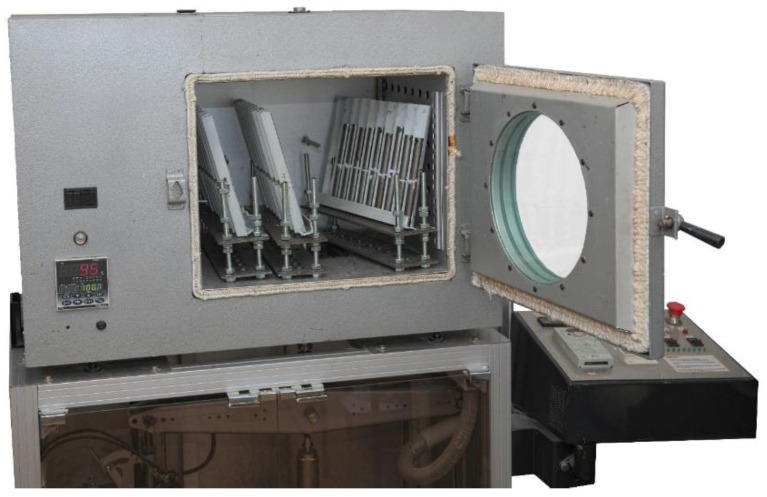
Positioning brackets for the correct orientation of the bonded specimens in the heating chamber.

Using the above adhesives, cylindrical steel specimens (S235JR/USt37-2/A283 Gr.C) with dimensions of ⌀20 × 100 mm were butt jointed. The faces of the specimens were prepared for bonding in accordance with PN-EN 13887:2005 (Adhesives for structural joints—Guidelines for preparing the surfaces of metals and plastics before bonding) in a turning operation with the surface roughness. The average of the profile height deviations from the mean line Ra = 3.4 ± 0.6 μm, and the ISO roughness grade: N8-N9 [30]. To minimise the impact of corrosion processes on the properties of the joint, the time from finishing the surface treatment to bonding was no more than 2 h. Maintaining a precise fit and parallelity of the bonded surfaces required the use of a custom-built bonding stand and special U-joint fixtures. The series for which the joints were planned to be heat cured were placed in an oven controlled by a Shimaden FP93 digital controller connected to a PC (Figure 2).

### 2.2. Mechanical Testing

**Figure 3:**
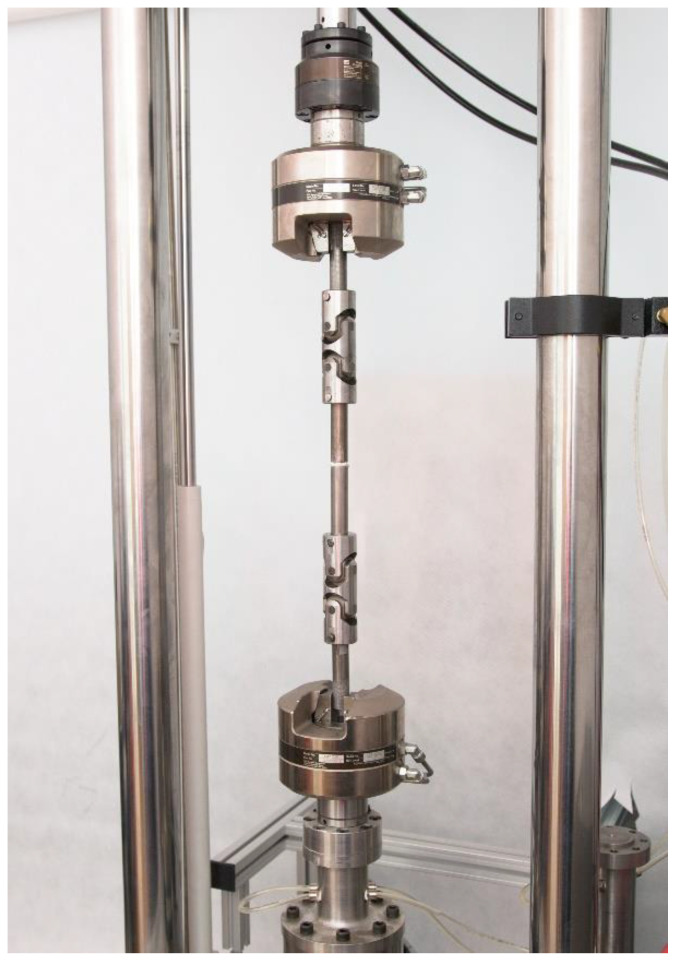
The adhesive specimen in the jointed fixture of the testing machine.

The adhesive joints were tested for axial tensile strength, which corresponds to pulling straight, in-plane, and away from the adhesive bond. For this purpose, the joints were fixed in the clamps of the testing machine (Figure 3) using U-joint grips and then pulled at a constant speed of 4 mm/min while recording the time, deformation and value of the tensile force. The tests were carried out in two series for each of the prepared combinations, i.e., at an ambient temperature of 20 °C and, using the heating chamber of the testing machine, at an elevated temperature (50 °C for the joints made with E57/PAC adhesive and 70 °C for Hysol 9492). At the end of each test run, the failure mode of the individual joints was recorded according to the EN ISO 10365:2022 standard (Adhesives—Designation of main failure patterns) [20].

### 2.3. Statistical Analysis

Statistical analysis is necessary to answer questions regarding actual batch-to-batch comparison, and it is only in this way that it is possible to estimate changes in strength due to variations in adhesive preparation accuracy and other parameters. If multiple averages need to be compared, simple statistical tests (Student’s *t*-tests) do not fulfil their purpose, as, the higher the number of averages being compared, the greater the risk of error for such comparisons. The correct method, the results of which are described below, involves isolating homogeneous groups of results, between which the differences are not statistically significant (at the assumed significance level of α = 0.05). A detailed description of the mathematical operations carried out to test the comparison of multiple batches is given, among others, in: [44,45,46]. The assumptions preceding the applicability of the method (normal distribution of results within the series, equality of variance within groups) were tested and confirmed. Tukey’s statistic tests were used in a version for different numbers of samples. This approach was chosen due to the unequal numbers of samples within the individual series, resulting, for example, from the exclusion of samples whose values were characterised by coarse errors, i.e., they differed significantly from other measurement results of the same quantity. The aforementioned test is one of several available in the software used in the analyses described in this paper (Tibco Statistica 13). Other tests of this type include Scheffé’s test, Newman and Keuls, Duncan’s test and Fisher’s NIR [44].

### 2.4. Mathematical Modelling

In order to gain a better understanding of the relationship between the degree of quantitative inaccuracy of the resin/hardener ratio, heat curing and the temperature of the working joint and its strength, further analysis was carried out to match typical mathematical models to the results obtained. A detailed description of the technique of regression modelling carried out in the paper is presented in: [47,48]. Given the nature, process and arrangement of the results obtained, simple models were tested: a linear model (which can perform well within the range of inaccuracy of the ratios studied in this paper) and a polynomial model of the 2nd, or possibly 4th, degree (which, as polynomial models of an even degree, will correspond to the course of a function whose extremes will tend towards zero from above, as is to be expected in a complete approach to the problem). For each fit, the R^2^ value was estimated, i.e., the coefficient of determination, which is a description of the quality of the fit of the model under test to the data obtained. The degree of fit of the selected models was examined.

## 3. Results

**Figure 4:**
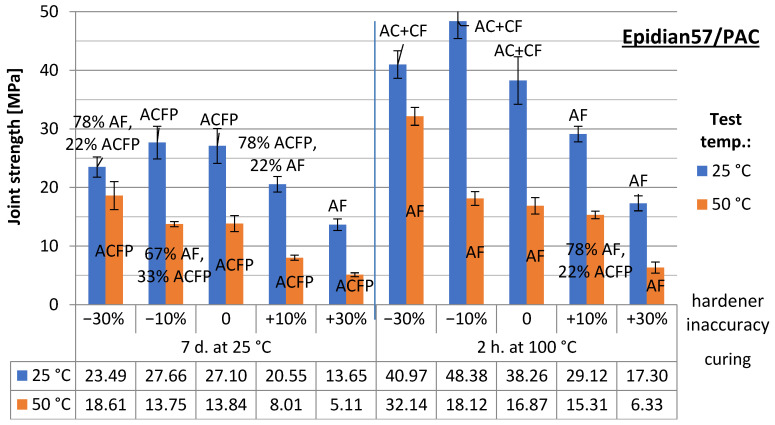
Test results for the adhesive joint strength made with Epidian57/PAC.

**Figure 5:**
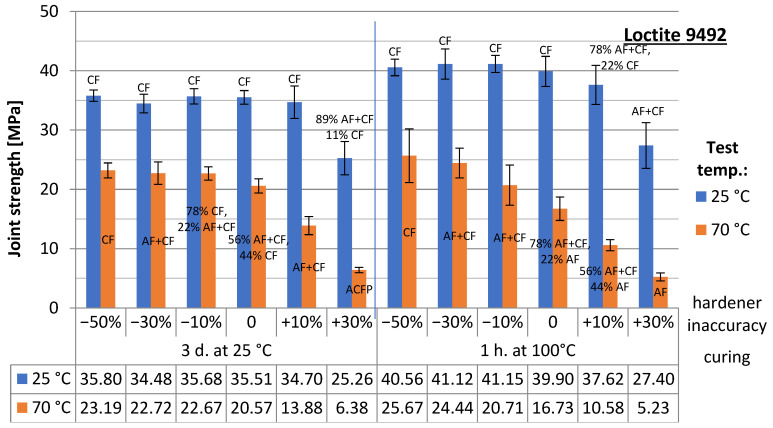
Test results for adhesive joint strength made with Hysol 9492.

**Figure 6:**
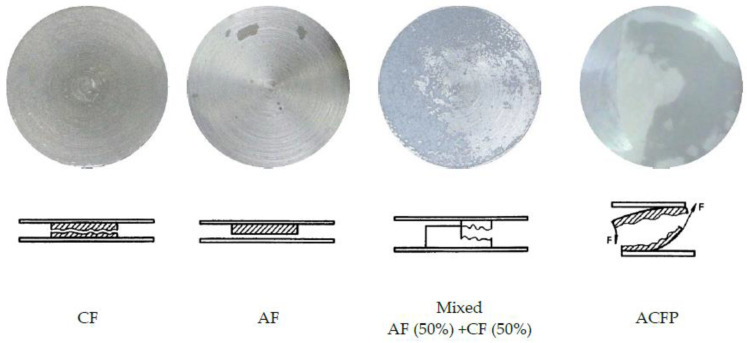
Main failure patterns of the tested adhesive joints [20].

The results of the strength tests are presented in Figure 4 (samples joined with Epidian 57/PAC adhesive) and Figure 5 (Hysol 9492). The results of the joints, grouped according to the same curing conditions (heat- and non-heat cured) and divided into two series—depending on the test temperature (ambient and elevated temperature) and taking into account the degree of the under/over-hardener in the adhesive formulation—are summarised there. The error bars indicating the standard deviation of the results for the two series as well as the failure pattern of the joint according to ISO 10365:2022 (Adhesives—Designation of main failure patterns) [20] are included in both diagrams: AF—adhesive failure, CF—cohesive failure, AC + AF—adhesive-cohesive failure, ACFP—adhesive failure with adhesive layer detachment. An example of the specimens after the destruction of the adhesive bond is shown in (Figure 6 [20]). In the case of the mixed-failure character, the percentage of each type within the reported series was additionally given. Below the plots are tables indicating the average strength values of each series.

From all the results obtained, the trend towards joint degradation with an excess of the hardener is most evident. The deficiency is considerably less unfavourable, even at high hardener deficiencies of 30–50%. The hardener, as a crosslinking reaction agent, allows it to continue even at lower dosage levels. The crosslinking reaction continues, producing an adhesive material with reasonably good performance as an adhesive. The analysis of failure models for joints made with Epidian 57/PAC adhesive shows that, often, the lesser the hardener is, the more important the cohesive nature of the failure becomes, especially for joints tested at ambient temperature. Operating the joints at elevated temperatures leads to a decrease in adhesion at the interface between the adhesive phases and the bonded material, so the joints are destroyed more often due to the detachment of the adhesive from the sample surface. Joints made with Hysol 9492 exhibit very strong adhesion at room temperature. Most samples show a cohesive failure pattern. Only a large excess of the hardener leads to a weakening of the adhesive structure and an increase in the proportion of cohesive failure (regardless of whether the joints were heat cured or cured at room temperature during the crosslinking stage).

Table 1 and Table 2 present the results of the statistical analysis, i.e., the obtained groups of results of homogeneous joint strengths made with Hysol 9492 adhesive, grouped according to the joint curing conditions and taking into account the joint test temperature. The groups include series marked in the tables with the symbol X, for which, with the adopted significance level α = 0.05, there are no statistically significant differences.

The statistical analysis clearly shows and confirms what the analysis of the average strength results initially indicated. An excess hardener almost always leads to a degradation of the strength characteristics of the joints. This is particularly noticeable when the joint is used at elevated temperatures. However, a deficiency of the hardener, in terms of the strength of the joint, can slightly, but in some cases significantly, increase the strength.

Table 3 and Table 4 show similar results for joints made with Epidian 57/PAC.

In the case of joints made with Epidian 57/PAC, the strength changes are of a similar nature to those observed for joints made with the first adhesive analysed. An excess of the hardener leads to strength degradation, especially in joints tested at elevated temperatures. At the same time, similarly, a shortage of the hardener allows stronger joints to be obtained.

On the basis of the data obtained, an attempt was made to fit them into simple mathematical models. Given the nature of the problem under analysis, it appears that the best fit will be a second-order polynomial model. This choice is due to the fact that there is a closed bounding interval for the hypothetical joint inaccuracy: from −100% (i.e., resin without hardener) to +100% (hardener alone without resin), with an extreme (maximum) somewhere in the middle. Of course, the range investigated in this work is limited on the practical side, from −50%/−30% to +30%. For this reason, an additional attempt was made to fit a linear model within this range. Table 5 and Table 6 present the results of the mathematical analyses of the regression models, together with the values of the individual coefficients of determination that indicate the quality of a given model’s fit to the data (R^2^).

An analysis of the results above shows that, in most cases, the linear model does not very accurately describe the variation in strength from the adhesive inaccuracy. The fit coefficients R2, depending on the combination of joint curing conditions and test temperatures, varied between 0.29 and, in some cases, even up to 0.91. However, the linear model more accurately described only joints tested at elevated temperatures (0.67–0.91), while the same joints tested at ambient temperature obtained values in the range of 0.29–0.61. In these cases, second-order (quadratic) polynomial models often proved to be significantly more accurate (0.51–0.77), representing a coefficient gain in the range of 27 to 77%. Attempts were made to model with higher-order polynomials due to the physical nature of the experiment (even-order), but the increase in fitting accuracy was no longer so significant, as, for example, when analysing a 4th degree polynomial model, the increments averaged about 7% (9.5% for joints tested at 20 °C and 4.4% for those tested at an elevated temperature). However, it should be kept in mind that increasing the polynomial degree of the model, although it may lead to a better fit to the local data, will result in a high sensitivity of the model outside the range of the data used and is not necessarily more beneficial in a general sense.

**Figure 7:**
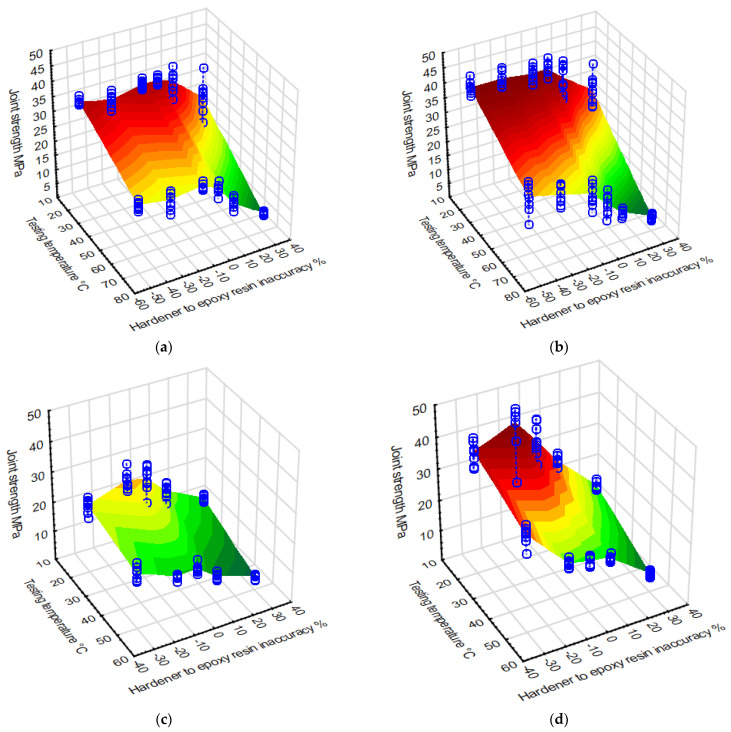
Strength of adhesive joints prepared using the presented adhesives crosslinked under different thermal conditions: (**a**) Hysol 9492 cured for 3 days at 20 °C, (**b**) Hysol 9492 cured for 1 h at 100 °C, (**c**) Epidian 57/PAC cured for 7 days at 20 °C, (**d**) Epidian 57/PAC cured for 2 h at 100 °C.

Finally, an attempt was made to model the area of change in the strength of joints made with the analysed adhesives, depending on the use of heat at the stage of crosslinking the adhesive material (forming the adhesive joint) and taking into account the thermal conditions of joint operation. Figure 7 shows “wafer” diagrams modelling the course of changes in joint strength depending on the inaccuracy of the share of the hardener in the adhesive composition and the joint test temperature, grouped according to the type of adhesive used and its curing conditions.

The above modelling results, in the form of the presented graphs, provide a comprehensive overview of the strength variations and the influence of two analysed factors: the degree of accuracy of the epoxy formulation and the joint test temperature. The three-dimensional structure of the presentation of the results obtained from destructive experimental tests, together with the modelled plane, also allow for the observation of the character of the transition of the area of the estimated joint strength at intermediate values of the operating temperature, which may be important when there is a need to estimate the strength at parameters with values in between those used for modelling.

## 4. Discussion

Adhesion engineering is directly related to chemistry, physics and mechanics; therefore, the analysis of adhesive bonding issues often involves topics from more than one of the aforementioned disciplines (multidisciplinary approach). In this paper, the focus is on the mechanical strength approach to the analysis of adhesive joints and the adhesives themselves, without touching on the chemical aspects in too much detail, which, in the longer term, may contribute interesting observations to the results of the paper. As described above, the final strength of structural adhesive joints has been shown to be relative to several factors, such as the state of preparation of the surfaces to be bonded [49,50], the type of adhesive used and its modifications [51], the curing conditions of the joint [52] and the operating conditions, among others.

Deviation from the stoichiometric amount of a curing agent has an impact on resin characteristics at the processing stage. Differences in the melt flow time and glass transition temperature (processability) of aerospace-grade resins and changes in ageing behaviour due to non-ideal DDS isomers, amine-to-epoxy (a/e) stoichiometric ratio material and process conditions have been found [53]. The predictive models were developed with the potential to provide the adaptive manufacturing of high-quality parts. The change in the characteristics of the cured products, e.g., among other things, the flexibility of materials based on the standard epoxy resin and the modified epoxy resin with different glycol molar ratios, was also analysed [54]. It has also been shown that samples with a low stoichiometric ratio have a predominantly branched molecular structure, whereas samples with a high stoichiometric ratio have a predominantly chain extension type structure. As a result, samples with a higher stoichiometric ratio increase the ductility of the materials, and the elongation at break increases, but the Young’s modulus decreases as the stoichiometric ratio increases [55]. The effect of the epoxy–amine ratio on the thermal properties, cryogenic mechanical properties and liquid oxygen compatibility of a phosphorus-containing epoxy resin was investigated, suggesting that reduced stiffness (resulting from a change in the epoxy–amine ratio) may be beneficial for improving the compatibility of the polymer with liquid oxygen [56]. A different study [57] analysed the mechanical properties as a function of the stoichiometry and chain structure of the components used. A good correlation was found between the toughness and ductility of these materials. The hardness behaviour was explained as a function of the homogeneity of the network and the extensivity of the segments forming the cross-linked structure. The tension and three-point bending of DGEBA-epoxy resin cured by DDM were also studied. As a result, the dependencies of the resin’s physical-mechanical properties (modulus of elasticity, strength, elongation at break, glass temperature) on the concentrations of the amine and epoxy groups ratio and the curing temperature were obtained [58]. In the same area, more practical cases were also investigated, e.g., epoxy resin glass fibre-reinforced composite. It was found that the epoxy resin: curing agent ratio did indeed influence both interfacial and thermal properties [59]. Interfacial Shear Stress (IFSS), using the test for the Thermal Mechanical Analyser (Araldite 506 epoxy resin and triethylenetetramine (TETA) hardener), was also analysed for fibre-reinforced polymers [60]. There are also known attempts at investigating the behaviour of a composite based on industrial hemp fibre and epoxy resin in terms of the ratio of the epoxy resin to the hardener. It was found that alkali treatment increased the interfacial shear strength, the composite tensile strength, the Young’s modulus and the elongation at break. The highest tensile strength was obtained with an epoxy-resin-to-curing-agent ratio of 1:1, while the best Young’s modulus was achieved with a resin-to-agent ratio of 1:1.2 [61].

The results obtained in the study presented in this paper confirm the influence of the inaccuracy of the amine-to-epoxy (a/e) stoichiometric ratio in the structure of adhesives on the strength of joints made with them. An excess of as little as 10% hardener was found to cause a statistically significant deterioration in strength (regardless of the type of adhesive tested), but, interestingly, a small deficiency of this hardener led to an increase in strength. A statistically significant increase in strength was shown to be particularly evident for joints tested at elevated temperatures, regardless of whether the adhesive was of a rigid or flexible type. By comparing the two adhesives tested, it can be seen that when the adhesive bonds are working at elevated temperatures, they are far more sensitive to changes in the epoxy-to-resin ratio than they are when working at ambient temperature. It is worth noting that the more rigid Epidian 57/PAC, when subjected to heat curing, showed less sensitivity to hardener excess, as it only registered a loss in strength characteristics at greater than 10% excess amine hardener. It should be remembered, however, that strength is one, but not the only, important property in the context of the operation of adhesive joints. In the characteristics of the final epoxy adhesives, the curing process is of key importance. The conditions during which the curing of the epoxy resin will take place will affect the material characteristics of the adhesive obtained, such as density [62], glass transition temperature [63,64], thermal behaviour [65] or the strength of the material itself [66], but also the quality of the adhesive effect to the material to be bonded [67,68]. Dramatic effects on the dynamic mechanical behaviour of flexible epoxy are shown, for example, in [69], where the dynamic mechanical behaviour of a new kind of flexible epoxy FE-1, which was crosslinked under four different thermal crosslink conditions, was examined. The results obtained in the research described in this paper confirm this phenomenon, with heat-cured joints, in most cases, being significantly stronger than unheated ones (by 30%, on average, when tested at ambient temperature and 15% when tested at elevated temperatures), as mentioned before. In one case, however, a deterioration in strength was observed after heat curing for joints made with the more rigid Hysol 9492 adhesive with an excessive proportion of a curing agent—only when tested at elevated temperatures (70 °C).

Despite the many benefits of epoxy resins and adhesives formed on their basis, one of their disadvantages is a certain sensitivity to operating conditions, precisely elevated temperatures [70,71,72,73,74,75], elevated pressures [76] and other interdependent factors, such as chemical agents typical of aerospace operations: water, jet fuel, hydraulic fluid and fuel additive [77,78]. The problem of the thermal degradation of epoxy adhesives has particularly been given much attention. Isotropic conductive adhesives (ICAs) (eco-friendly alternatives to lead solder in surface mount technology (SMT)) have been studied during two different environmental tests: a thermal cycle from −40 to 125 °C and a humid exposure of 85 °C at 85% RH. The development of some additional defects at the joint interface, such as microcracks and layers of Sn oxide, resulted in the interfacial degradation of the assembled chip components. [79]. The electrically conductive adhesives (ECAs), e.g., silver-epoxy ECAs, can also be affected due to environmental ageing (heat/humidity ageing (85 °C/85% relative humidity) and accelerated thermal cycling (−40 to 125 °C)), as it affects their electrical conductivity [80]. Due to the need for the joint to operate at a specific temperature, research was also carried out to analyse potential adhesives made of two compositions—one highly resistant to low temperatures and the other resistant to high temperatures—and to build models to estimate the strength of such a bi-component adhesive. Numerical analysis was conducted using finite element models to investigate the distribution of stresses in the mixed adhesive joint in order to find the optimal design for titanium/um/titanium and titanium/composite double lap joints. For the joint with non-similar adhesives, the mixed double adhesive joint was shown to produce an increased load capacity for the considered temperature range compared to the application of a high-temperature adhesive itself. [81]. As shown, the epoxy adhesives tested in this work also showed significant sensitivity to the elevated temperature conditions, which always weakened the joints analysed.

Further research in the topic is planned to analyse the influence of other parameters on the strength of adhesive joints, including: the method of surface preparation, the use of admixtures of materials that modify the properties of the adhesive, the degradation of joints due to aggressive environmental conditions and variable cyclic fluctuations in the temperature of the working environment of the joint [82,83,84,85]. To this end, statistical tools will be used to support mathematical modelling, and it is also planned to apply an analytical approach and use finite element numerical analysis software [86,87,88,89,90]. All this with the aim of obtaining even more durable and reliable adhesive joints that will be able to complement but also compete with other types of joints to an even greater extent.

## 5. Conclusions

The inaccuracy of the amine-to-epoxy stoichiometric ratio and the fact that heat is used to assist in curing the adhesive (resin cross-linking) are important factors in the design of adhesive joints. By using experimental tests on various types of adhesives and valid statistical methods (post-hoc tests), the influence of the variation in the above parameters was assessed, and a statistically significant variation in strength depending on the parameters analysed was demonstrated. As little as a 10% excess hardener, in the case of a more rigid adhesive, will, in most cases, lead to a significant deterioration in the strength of the joint. The flexible Hysol 9492, on the other hand, is resistant to up to a 30% excess hardener. On the other hand, a shortage of the hardener either did not adversely affect the strength of the joints or even led to an improvement. However, one should be cautious, as this may be apparent, and the modification of the adhesive may be followed by other negative changes of a non-strength nature.

It was shown that the heat curing operation at the stage of forming the butt joint leads to an increase in its static strength at ambient temperature, as well as in the case of joints made with adhesives with a deviation from the initial resin/hardener ratio considered optimal. It has been proven, however, that the heat curing operation cannot be regarded as universally improving the strength of the joint in all operational cases of the joint (e.g., at elevated temperatures of joint operation).

A reasonably good fit was achieved between simple mathematical models and the results obtained, which makes it possible to use them to predict other intermediate cases of variation in the parameters analysed, without the need for further destructive tests on adhesive joints. As shown in the paper, the second-order polynomial model was sufficient to describe the variation in strength from the inaccuracy of the epoxy/amine hardener ratio quite well. In addition, higher even-order models were tested, but it was not considered that a slight increase in fit would justify the use of these types of curves.

In the research described in this paper, the adhesive joint was analysed in one of several possible joint loading arrangements, i.e., butt joint, axially tensile. Classical test methods for adhesives and adhesive joints often also pay a lot of attention to lap, shear-operated joints, as this is one of the more favourable loading options for adhesive joints. It thus seems reasonable to continue the tests that have been undertaken for other joint conditions to see whether the observed patterns will be confirmed in other cases. In addition, it is worth including chemical and thermal analyses in the study, which could show in a practical way how an inaccurately made adhesive cross-links and which cross-linking state corresponds to which strength characteristics of the adhesive and the joints made with it.

## Figures and Tables

**Table 1:** Homogeneous groups (1-2, 1-2-3) of strength at 20 °C for joints made with Hysol 9492.

Curing Conditions	3 Days in 20 °C			1 h in 100 °C			
Hardener Inaccuracy	Mean Strength (MPa) at 20 °C	1	2	Mean Strength (MPa) at 20 °C	1	2	3
−50%	35.81	X		41.06	X		
−30%	33.46	X		41.12	X		
−10%	35.68	X		39.89	X	X	
**0%**	**35.51**	**X**		**39.90**	**X**	**X**	
10%	33.82	X		35.71		X	
30%	25.44		X	28.50			X

**Table 2:** Homogeneous groups (1-2-3-4, 1-2-3-4-5) of strength at 70 °C for joints made with Hysol 9492.

Curing Conditions	3 Days in 20 °C					1 h in 100 °C					
Hardener Inaccuracy	Mean Strength (MPa) at 70 °C	1	2	3	4	Mean Strength (MPa) at 70 °C	1	2	3	4	5
−50%	23.18	X				25.67	X				
−30%	21.60	X	X			23.09	X	X			
−10%	22.67	X	X			19.93		X			
**0%**	**20.12**		**X**			**14.77**			**X**		
10%	13.49			X		10.10				X	
30%	6.64				X	5.25					X

**Table 3:** Homogeneous groups (1-2-3-4, 1-2-3-4) of strength at 20 °C for joints made with Epidian 57/PAC.

Curing Conditions	7 Days in 20 °C					2 h in 100 °C				
Hardener Inaccuracy	Mean Strength (MPa) at 20 °C	1	2	3	4	Mean Strength (MPa) at 20 °C	1	2	3	4
−30%	22.88		X	X		39.50		X		
−10%	27.39	X				47.21	X			
**0%**	**25.95**	**X**	**X**			**37.29**		**X**		
10%	19.97			X		29.12			X	
30%	13.65				X	17.30				X

**Table 4:** Homogeneous groups (1-2-3-4, 1-2-3-4) of strength at 50 °C for joints made with Epidian 57/PAC.

Curing Conditions	7 Days in 20 °C					2 h in 100 °C				
Hardener Inaccuracy	Mean Strength (MPa) at 50 °C	1	2	3	4	Mean Strength (MPa) at 50 °C	1	2	3	4
−30%	18.10	X				31.41	X			
−10%	13.71		X			18.13		X		
**0%**	**13.84**		**X**			**16.87**		**X**	**X**	
10%	9.09			X		15.55			X	
30%	4.79				X	6.39				X

**Table 5:** Fitting the strength variation curve against the inaccuracy of the adhesive preparation to the linear model: Strength = a + b · (inaccuracy).

Adhesive	Test Temperature	Curing Conditions	a	b	R^2^
H9492	20 °C	1 h at 100 °C	36.43	−14.50	0.473
H9492	20 °C	3 d at 20 °C	32.53	−9.28	0.291
H9492	70 °C	1 h at 100 °C	14.22	−26.73	0.804
H9492	70 °C	3 d at 20 °C	16.36	−19.28	0.673
E57 + PAC	20 °C	2 h at 100 °C	33.61	−41.16	0.611
E57 + PAC	20 °C	7 d at 20 °C	22.13	−17.17	0.380
E57 + PAC	50 °C	2 h at 100 °C	17.67	−38.82	0.909
E57 + PAC	50 °C	7 d at 20 °C	11.97	−23.03	0.888

**Table 6:** Fitting the strength variation curve with respect to the inaccuracy of the adhesive preparation to a second-order polynomial model: strength = a + b·(inaccuracy) + c·(inaccuracy)^2^.

Adhesive	Test Temperature	Curing Conditions	a	b	c	R^2^
H9492	20 °C	1 h at 100 °C	38.48	−21.99	−35.16	0.651
H9492	20 °C	3 d at 20 °C	34.34	16.44	−31.94	0.514
H9492	70 °C	1 h at 100 °C	15.28	−30.71	−18.37	0.822
H9492	70 °C	3 d at 20 °C	18.69	−28.51	−41.56	0.874
E57 + PAC	20 °C	2 h at 100 °C	37.76	−41.16	−103.68	0.774
E57 + PAC	20 °C	7 d at 20 °C	24.94	−17.96	−75.51	0.697
E57 + PAC	50 °C	2 h at 100 °C	16.70	−38.82	24.35	0.924
E57 + PAC	50 °C	7 d at 20 °C	12.29	−22.98	−7.74	0.892

## Data Availability

The data presented in this study are available from the corresponding authors upon request.
